# Acupuncture-related brain network findings across clinical stages of the Alzheimer’s disease continuum: a critical review of functional MRI evidence

**DOI:** 10.3389/fneur.2026.1977763

**Published:** 2026-10-28

**Authors:** Xue Bai, Yahong Ji, Hengyu Ruan, Wenhao Zhang, Yunlong Ma, Xiaoxu Jiang, Xiaohui Liu, Feng Wang

**Affiliations:** 1 Graduate School of Heilongjiang University of Chinese Medicine, Harbin, China; 2 Division of CT and MRI, First Affiliated Hospital, Heilongjiang University of Chinese Medicine, Harbin, China

**Keywords:** acupuncture, Alzheimer’s disease, brain networks, functional connectivity, functional MRI, mild cognitive impairment, subjective cognitive decline

## Abstract

Brain network abnormalities are observed across the Alzheimer’s disease (AD) continuum, but the relationship between clinical stage, acupuncture-related functional brain changes, and cognitive outcomes remains unclear. This review examined clinical blood oxygen level-dependent functional MRI studies of acupuncture in subjective cognitive decline (SCD), mild cognitive impairment (MCI, including amnestic MCI [aMCI]), and AD dementia. PubMed, Web of Science Core Collection, and China National Knowledge Infrastructure (CNKI) were searched for relevant studies. The evidence was synthesized narratively by clinical stage, with attention to study design, acupuncture protocols, control conditions, imaging methods, brain–cognition associations, biomarker status, and possible cohort overlap. In SCD, the limited available evidence mainly involved changes in regional spontaneous activity, hippocampal connectivity, and network organization. In MCI, including aMCI, reported changes involved memory- and limbic-related regions, DLPFC–ACC connectivity, and locus coeruleus-related pathways. In AD dementia, reported changes mainly involved the hippocampus and posterior medial memory-related regions, with earlier studies mainly examining short-term responses and two longitudinal cohorts assessing changes over a treatment course. Across the reviewed literature, findings in MCI, including aMCI, involved a broader range of brain regions and networks, whereas those in AD dementia were more often centered on the hippocampus and posterior medial memory-related regions. These differences are difficult to attribute to clinical stage alone, given substantial differences across studies in acupuncture protocols, control conditions, imaging methods, sample size, biomarker confirmation, and follow-up. Larger longitudinal studies with biomarker confirmation where possible and complementary imaging modalities are needed to determine whether the observed differences are related to clinical stage and how the imaging changes relate to cognitive outcomes.

## 1 Introduction

Alzheimer’s disease (AD) develops over many years, with amyloid-β and tau pathology, changes in brain function, and cognitive decline appearing before dementia becomes clinically evident (1, 2). Although current diagnostic frameworks increasingly incorporate biomarkers into the definition and staging of AD (3), most acupuncture-related BOLD-fMRI studies still rely mainly on clinical and neuropsychological criteria, with limited biomarker confirmation. We therefore organize the evidence by the clinical categories used in these studies: SCD, MCI (including amnestic MCI [aMCI], a memory-predominant subtype), and AD dementia (4–7).

Acupuncture has been explored as a non-pharmacological approach to cognitive impairment (8, 9). With the increasing use of BOLD-fMRI, studies have examined acupuncture-related changes in local spontaneous activity, functional connectivity, and large-scale network organization (10–12). Acupuncture neuroimaging reviews have reported changes in the hippocampus and other cognition-related brain regions and networks (13, 14). The default mode network (DMN), salience network (SN), and frontoparietal network (FPN) are also affected across the AD continuum (15, 16), providing a relevant context for interpreting acupuncture-related BOLD-fMRI findings.

Previous reviews have summarized acupuncture-related neuroimaging findings in AD and MCI separately (13, 14), but evidence across SCD, MCI, and AD dementia has not been considered together. Considering these clinical categories within a common framework may help determine whether the reported differences are related to clinical stage or to differences in acupuncture protocols, control conditions, study design, and imaging methods, and how these imaging findings relate to cognitive outcomes. Biomarker status, where available, may further help place these findings in the context of underlying AD pathology. In this review, we synthesize clinical BOLD-fMRI evidence across SCD, MCI (including aMCI), and AD dementia, considering imaging findings alongside cognitive outcomes, biomarker status, and possible sample overlap to clarify the patterns observed across these clinical categories and the factors that limit their interpretation. The overall framework of the review is shown in Figure 1.

**Figure 1:**
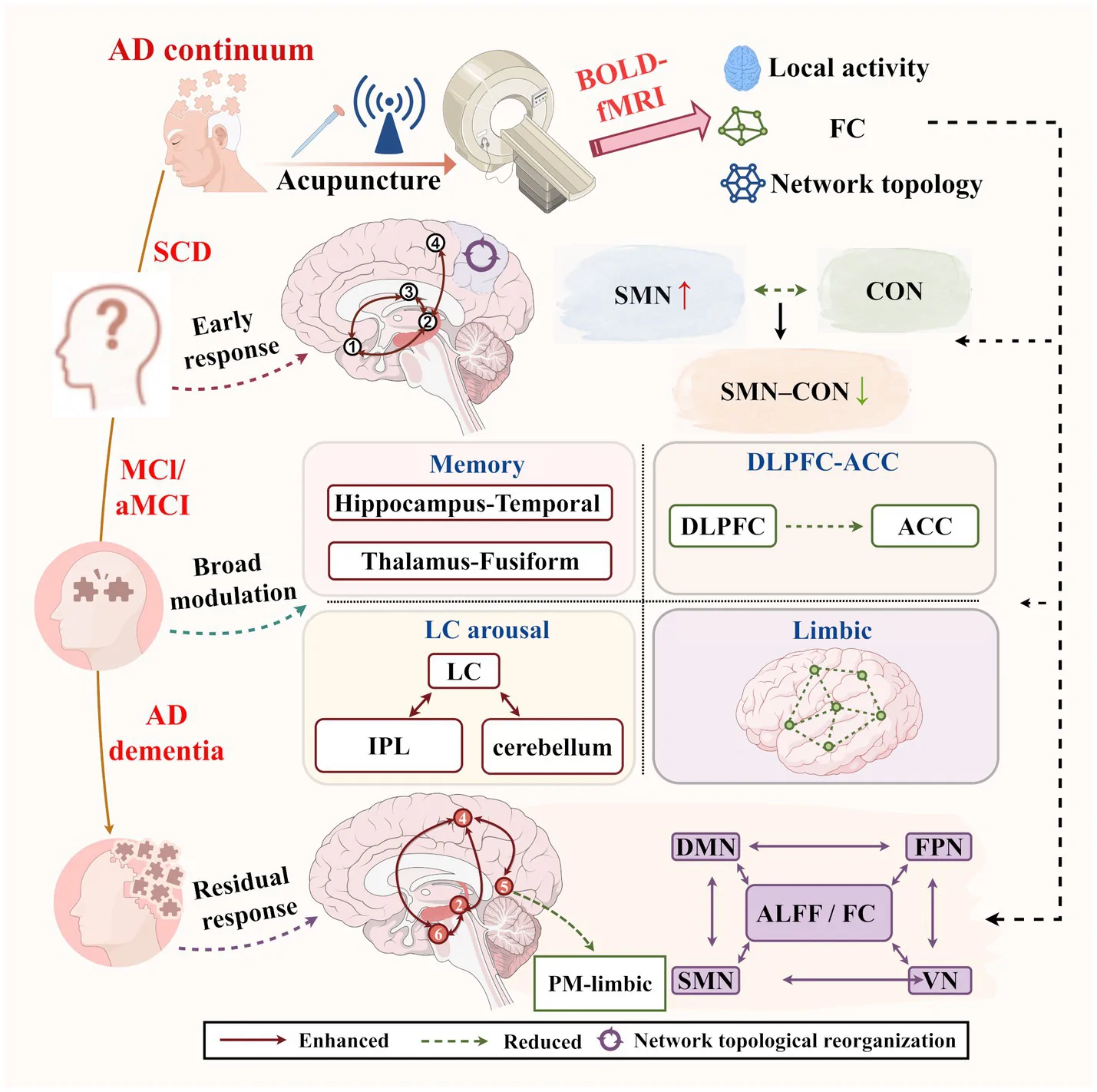
Schematic summary of reported acupuncture-related BOLD-fMRI findings across the clinically defined Alzheimer’s disease continuum. In subjective cognitive decline, the available studies report changes in regional spontaneous activity, hippocampal connectivity, and broader network organization. In mild cognitive impairment, including amnestic mild cognitive impairment, reported findings involve memory- and limbic-related regions, DLPFC–ACC connectivity, and locus coeruleus-related pathways. In Alzheimer’s disease dementia, reported findings more often involve the hippocampus and posterior medial memory-related regions, with additional changes in other large-scale networks. Numbers in the schematic indicate representative brain regions: ① temporal lobe, ② hippocampus, ③ insula, ④ posterior cingulate cortex/precuneus, ⑤ retrosplenial cortex, and ⑥ parahippocampus. Solid arrows indicate enhanced connectivity or activity, dashed arrows indicate reduced connectivity or activity, and circular arrows indicate network-topological reorganization. Created with Figdraw. AD, Alzheimer’s disease; SCD, subjective cognitive decline; MCI, mild cognitive impairment; aMCI, amnestic mild cognitive impairment; DMN, default mode network; FPN, frontoparietal network; SMN, sensorimotor network; CON, cingulo-opercular network; DLPFC, dorsolateral prefrontal cortex; ACC, anterior cingulate cortex; LC, locus coeruleus; IPL, inferior parietal lobule; PCC, posterior cingulate cortex; RSC, retrosplenial cortex; PM, posterior medial; VN, visual network. Schematic diagram showing reported acupuncture-related BOLD-fMRI findings across the clinically defined Alzheimer’s disease continuum. In SCD, the available studies show changes in regional spontaneous activity, hippocampal connectivity, and network organization. In MCI, including aMCI, reported findings involve memory- and limbic-related regions, DLPFC–ACC connectivity, and locus coeruleus-related pathways. In AD dementia, findings more often involve the hippocampus and posterior medial memory-related regions, with additional changes in other large-scale networks.

## 2 Literature search and evidence synthesis

PubMed, Web of Science Core Collection, and China National Knowledge Infrastructure (CNKI) were searched for relevant studies, with the search last updated on October 14, 2026. The search was structured around terms for the clinical population, acupuncture, and BOLD-fMRI, including “Alzheimer’s disease,” “subjective cognitive decline,” “mild cognitive impairment,” “amnestic mild cognitive impairment,” “acupuncture,” “electroacupuncture,” “BOLD-fMRI,” “resting-state fMRI,” “functional connectivity,” “effective connectivity,” “regional homogeneity,” “ALFF,” “brain network,” “default mode network,” and “hippocampus.” The search strategy was adapted to the syntax and fields of each database, with complete database-specific strategies provided in Supplementary Table S1. Reference lists of relevant studies and reviews were also screened. Original clinical studies were considered for the primary synthesis if they included participants with SCD, MCI (including aMCI), or AD dementia, used manual acupuncture or electroacupuncture as an intervention or stimulation condition, and reported acupuncture-related BOLD-fMRI outcomes involving regional spontaneous activity, functional or effective connectivity, or brain-network organization. Both single-session stimulation and longitudinal intervention studies were eligible. For studies combining acupuncture with another treatment, the acupuncture-related effect had to be distinguishable from the co-intervention. Studies involving only healthy participants or animal models, studies without separately reported data for the relevant patient group, and studies reporting only structural or other non-BOLD neuroimaging findings were not included as primary evidence. Conference abstracts, dissertations, and theses were not included. Studies limited to task-evoked activation without regional spontaneous-activity, connectivity, or network-level outcomes were considered only as contextual evidence where relevant. Two reviewers independently screened titles and abstracts and assessed potentially eligible reports in full text; disagreements were resolved through discussion.

The included studies were synthesized narratively by clinical stage, taking into account study design, sample size, acupuncture and control procedures, imaging methods, follow-up, cognitive outcomes, biomarker status, brain–cognition associations, and possible cohort overlap. When multiple reports appeared to arise from the same or overlapping participant cohort, they were retained if they provided distinct imaging analyses but were linked and were not treated as independent participant-level evidence. These characteristics are summarized in Tables 1, 2 and were considered when interpreting the imaging findings. No formal risk-of-bias or certainty-of-evidence tool was used; instead, the main methodological limitations of individual studies were considered as part of the narrative synthesis. Because the evidence base was small and heterogeneous, no quantitative pooling was performed.

**Table 1:** Characteristics and critical appraisal of clinical acupuncture-related BOLD-fMRI reports included in the primary synthesis.

Report(s)	Stage/diagnosis	Design and sample	Acupuncture intervention and control	BOLD-fMRI measure(s)	Main imaging findings	Evidence note
Zhang et al. (31)	SCD	Longitudinal controlled study; 16 SCD participants assigned to acupuncture (n = 8) or no-treatment control (n = 8); pre/post rs-fMRI.	Manual acupuncture twice weekly for 12 weeks; no-treatment control.	ReHo.	Compared with the no-treatment group, acupuncture was associated with decreased ReHo in the prefrontal cortex.	Very small study with a no-treatment rather than sham-acupuncture control; AD biomarker confirmation was not reported.
Wang et al. (32)^§^	SCD	Longitudinal sham-controlled RCT; 36 acupuncture and 36 sham participants; pre/post rs-fMRI.	Multi-point acupuncture, with electroacupuncture applied to GV20 (Baihui) and GV24 (Shenting); twice weekly for 12 weeks; nonpenetrating sham-acupuncture control.	Hippocampal FC.	Compared with sham acupuncture, acupuncture was associated with increased hippocampal FC involving the PCC/precuneus, insula, and temporal regions.	Longitudinal sham-controlled RCT; structural and diffusion MRI findings were treated only as related non-BOLD evidence; AD biomarker confirmation was not reported.
Zhou et al. (33)^§^	SCD	Secondary whole-brain network analysis of the Wang et al. (32) RCT; 66 participants.	Same 12-week acupuncture regimen; sham control.	Whole-brain FC; graph theory.	Two within-SMN FCs increased, whereas three SMN–CON FCs decreased; left parietal nodal efficiency increased and right parietal betweenness decreased, based on significant group-by-time effects; interpreted as secondary evidence from the same RCT.	Secondary analysis of the same SCD RCT as Wang et al. (32); informative for network-level changes but not independent evidence.
Feng et al. (39)^*^	MCI	Acute single-session stimulation study; 12 MCI patients and 12 healthy controls.	Manual acupuncture at KI3; deep versus superficial needling; healthy-control comparison.	Whole-brain FC.	Enhanced post-stimulus functional connectivity involving the hippocampus, thalamus, and fusiform gyrus; deep acupuncture produced stronger changes than superficial acupuncture.	Small single-session study; findings mainly reflect immediate brain responses after KI3 stimulation.
Bai et al. (40)^*^	MCI	Acute needling-depth study; 12 MCI patients and 12 healthy controls.	Manual acupuncture at KI3 (Taixi); deep versus superficial needling.	Graph theory; small-world properties; nodal centrality.	Deep needling modulated abnormal small-world properties and increased nodal centrality in regions including the hippocampus, postcentral cortex, and ACC; superficial needling showed weaker effects.	Related to the same or substantially overlapping early KI3 cohort; retained because it provides a distinct network-topology analysis.
Chen et al. (38), KI3^*^	MCI	Acute single-session stimulation study; 12 MCI patients and 12 healthy controls.	Manual acupuncture at KI3; deep versus superficial needling; healthy-control comparison.	Multivariate Granger causality; EC.	Deep needling was associated with broader directed network changes, with the DLPFC and hippocampus emerging as prominent EC nodes.	Small single-session effective-connectivity study; clinical relevance and longer-term effects remain unclear.
Chen et al. (43), GV26^¶^	MCI	Acute single-session pre/post study; 6 MCI patients.	Single-session manual acupuncture at GV26 (Shuigou); resting-state scans before acupuncture and during needle retention.	Resting-state FC.	During needle retention, altered connectivity involved the IPL and prefrontal regions, including the DLPFC, aPFC, and VMPFC.	Very small uncontrolled acute study; appears to share the same participant cohort as Xu et al. (44), but reports connectivity during needle retention.
Xu et al. (44)^¶^	MCI	Acute single-session post-acupuncture study; 6 MCI patients.	Single-session manual acupuncture at GV26 (Shuigou); resting-state assessment after needle withdrawal.	Resting-state FC.	After needle withdrawal, IPL connectivity decreased, whereas connectivity involving the left DLPFC, bilateral frontal regions, and aPFC increased.	Very small uncontrolled acute study; appears to share the same six-patient cohort as Chen et al. (43), GV26, but reports the post-acupuncture resting period.
Liu et al. (41)^*^	MCI	Acute pre/post acupuncture study; 12 MCI patients and 12 healthy controls.	Manual acupuncture at KI3 (Taixi); pre- and post-acupuncture resting-state assessment.	ReHo.	Regional ReHo changes were observed after acupuncture; however, repeated-measures ANCOVA showed no significant acupuncture main effect or group × acupuncture interaction after multiple-comparison correction.	Small acute study from the same or substantially overlapping KI3 series; corrected analyses did not demonstrate a significant acupuncture or interaction effect.
Jia et al. (42)	MCI	Acute real-versus-sham acupoint study; 8 MCI patients and 15 healthy controls were reported as recruited.	KI3 (Taixi) versus a nearby sham point.	ALFF.	Direct KI3–sham comparison in MCI showed higher ALFF in the right superior temporal and cingulate gyri and lower ALFF in the left middle frontal gyrus; differences were limited to a few regions.	Very small acute study; participant accounting was internally inconsistent across the article, and the sham point was anatomically close to KI3.
Tan et al. (26)	aMCI	Randomized longitudinal study; 32 aMCI participants assigned to verum or sham-acupoint acupuncture.	EX-HN1, EX-HN3, PC6, KI3, ST40, and LR3 versus related sham acupoints; five sessions/week for 4 weeks.	Resting-state brain-network connectivity.	After verum acupuncture, connectivity increased among cognition-related regions including the insula, DLPFC, hippocampus, thalamus, inferior parietal lobule, and ACC; the insula, DLPFC, and hippocampus were prominent hubs.	Small longitudinal sham-acupoint-controlled study; no follow-up beyond the treatment period was reported.
Li et al. (46)	aMCI	Randomized longitudinal study; 28 aMCI patients and 50 healthy controls at baseline; three follow-up aMCI imaging datasets were excluded for excessive motion.	Manual acupuncture at bilateral LI4 (Hegu) and LR3 (Taichong); three sessions/week over 6 months; no-intervention aMCI control.	Hippocampal seed-based FC.	Compared with the control group, acupuncture was associated with increased right hippocampal FC with the right ITG and left MTG. Lower baseline right hippocampus–ITG FC was associated with greater subsequent memory change.	No sham-acupuncture control. Regular rivastigmine use was an eligibility criterion, but whether medication was continued throughout the study was not clearly reported.
Zhang et al. (47)	aMCI	Randomized, controlled, single-blind longitudinal study; 46 aMCI participants randomized to verum or sham acupuncture.	Verum versus sham acupuncture; 24 sessions, three times/week for 8 weeks.	ReHo; DLPFC seed-based FC.	Group × time effects included increased left DLPFC ReHo, increased left DLPFC–precuneus FC, and decreased left DLPFC–inferior temporal gyrus FC after verum relative to sham acupuncture. Change in left DLPFC ReHo correlated with change in MoCA.	Sham-controlled longitudinal study with a modest sample; AD biomarker confirmation was not reported.
Xu et al. (49)	aMCI	Randomized longitudinal study; 80 aMCI patients assigned to true acupuncture (n = 40) or sham acupuncture (n = 40); pre/post fMRI.	Acupuncture at Siguan points versus sham acupuncture.	ALFF; fALFF; ReHo.	After true acupuncture, ALFF increased in the left precentral gyrus and left cuneus and decreased in the right fusiform gyrus; fALFF increased in the left postcentral gyrus and right middle temporal gyrus; ReHo increased in the right inferior temporal gyrus.	Sham-controlled longitudinal study; imaging findings were mainly reported as within-group pre/post changes, limiting interpretation of acupuncture-specific effects.
Yin et al. (45), CTM	aMCI	Randomized controlled pilot study; 40 aMCI participants randomized to acupuncture or wait-list control.	Multi-point manual acupuncture including GV20, GV24, Anmian, HT7, and KI3; twice weekly for 12 weeks; wait-list control.	ReHo.	Compared with the wait-list group, acupuncture was associated with ReHo changes in the left medial orbital frontal gyrus, left middle cingulate cortex, and left superior medial frontal gyrus; imaging findings were examined together with cognitive and multi-omics measures.	Small pilot RCT integrating BOLD-fMRI with gut microbiota and serum metabolomics; the wait-list control does not account for treatment contact or expectation.
Yin et al. (48), JEBM	aMCI	Longitudinal RCT; 72 aMCI participants randomized to acupuncture (n = 36) or wait-list control (n = 36); 20 cognitively normal participants were additionally included as a reference group.	GV24, GV20, Anmian, HT7, and KI3; twice weekly for 12 weeks; wait-list control.	ReHo; seed-based FC; EC.	Compared with the wait-list group, acupuncture reduced abnormal left DLPFC ReHo and DLPFC–ACC FC; decreased DLPFC-to-ACC EC was associated with change in ADAS-Cog scores.	Longitudinal clinical-imaging study; the wait-list control limits interpretation of acupuncture specificity.
Han et al. (50)	aMCI	Non-randomized longitudinal controlled study; 35 aMCI participants received true-acupoint acupuncture and 15 received sham-acupoint acupuncture, with 41 healthy controls.	Yuan-Luo Tongjing acupuncture once daily, 6 days/week, for two 4-week courses with a 2-week interval; sham-acupoint control.	Limbic-system FC.	Within the true-acupoint group, FC between multiple limbic seed regions and frontal, cingulate, inferior parietal, and precuneus regions predominantly decreased after treatment; most pre/post changes in the sham-acupoint group did not survive FWE correction.	Non-randomized study with imbalanced treatment groups and baseline FC differences between the true- and sham-acupoint groups; these features limit causal interpretation. AD biomarker confirmation was not reported.
Hu et al. (53)	aMCI	Randomized sham-controlled longitudinal study; 50 participants were included in the final fMRI analysis (26 verum acupuncture, 24 sham acupuncture).	Standardized multi-point manual acupuncture; 30 min/session, three times/week for 8 weeks; superficial non-acupoint sham without manipulation or de qi.	LC-seeded FC.	Group × time effects involved increased left LC–left inferior parietal lobule FC and right LC–right posterior cerebellum FC after verum acupuncture; baseline left LC–inferior parietal lobule FC was associated with subsequent improvement in immediate recall.	Substantial attrition occurred between initial enrollment and the final imaging sample, and AD biomarker confirmation was not reported.
Zhang et al. (51)	aMCI	Randomized sham-controlled longitudinal study; 50 aMCI participants allocated to verum acupuncture (n = 24) or sham acupuncture (n = 26), with 34 healthy controls.	Verum versus sham acupuncture; 24 sessions over 8 weeks.	Hippocampal-subregion seed-based RSFC.	RSFC between the left hippocampal perceptual subregion and right middle frontal gyrus increased after verum acupuncture but not after sham acupuncture.	Brain–cognition associations were reported at baseline and post-treatment rather than as correlations between changes in connectivity and changes in cognition.
Qiu et al. (52)	aMCI	Randomized sham-controlled longitudinal study; 30 aMCI patients with comorbid insomnia were randomized to verum or sham acupuncture (15/group), with 15 healthy controls.	Multi-point manual acupuncture including GV20, EX-HN1, GV24, EX-HN3, CV6, CV4, LI4, HT7, ST36, ST40, SP6, GB39, KI3, and LR3; three times/week for 12 weeks; placebo-needle control.	ALFF; ReHo.	Significant group × time effects involved increased left calcarine ALFF and increased ReHo in cerebellar vermis VI after verum acupuncture, with opposite trends after sham acupuncture; changes in both imaging measures were associated with changes in MoCA.	Small RCT restricted to aMCI patients with comorbid insomnia; the comorbidity may influence cognition and BOLD-fMRI measures and limits generalizability to the broader aMCI population. AD biomarker confirmation was not reported.
Liang et al. (60)^‡^	AD dementia	Acute stimulation study; 14 AD patients and 14 healthy controls enrolled; 9 AD patients and 11 controls remained after exclusion for excessive head motion.	Single-session manual acupuncture at LR3 and LI4; healthy-control comparison.	ICA-DMN; ROI-based FC.	Changes in DMN activity/connectivity were reported involving the PCC, precuneus, inferior parietal lobule, and middle temporal gyrus.	Small acute LR3/LI4 study with healthy-control comparison; findings mainly reflect immediate BOLD-fMRI responses.
Wang et al. (59)^‡^	AD dementia	Acute pre/post rs-fMRI stimulation study; 14 AD patients and 14 healthy controls.	Single-session manual acupuncture at LR3 and LI4; healthy-control comparison.	Hippocampal FC.	Increased hippocampal FC, including enhanced left hippocampus-right middle frontal gyrus connectivity.	Small acute pre/post study; findings mainly indicate short-term hippocampal connectivity changes.
Zheng et al. (62)^‡^	AD dementia	Acute stimulation study; 14 AD patients and 14 healthy controls.	Single-session manual acupuncture at LR3 and LI4; healthy-control comparison.	ALFF; seed-based FC.	Bidirectional ALFF changes and increased hippocampus-precentral gyrus FC.	Small acute LR3/LI4 study; lack of longitudinal follow-up limits interpretation.
Ji et al. (61)	AD dementia	Acute study; 14 AD patients and 14 healthy controls recruited; 12 AD patients and 12 controls included in the reported analysis.	Single-session manual acupuncture at LI4 and LR3; healthy-control comparison.	ICA of resting-state networks.	Acupuncture-related FC changes involved the left and right FPN, SMN, and visual network, with both increases and decreases reported across network regions.	Small acute network-level study. Although the stimulation paradigm resembled the earlier LR3/LI4 series, the analyzed sample differed demographically and was treated as a separate cohort.
Wang et al. (63)	AD dementia	RSC-seeded FC reanalysis of the Wang et al. (59) dataset; 14 AD patients and 14 healthy controls.	Single-session manual acupuncture at LR3 and LI4; healthy-control comparison.	RSC-seeded FC.	Bidirectional RSC-FC regulation: decreased RSC connectivity with the thalamus, PCC, SCG, OFC, and precuneus in AD patients but increased connectivity in healthy controls; specifically increased RSC–hippocampus and RSC–parahippocampal FC in AD patients.	Reanalysis of the Wang et al. (59) dataset; findings should therefore not be counted as independent participant-level evidence.
Fan et al. (64)^#^	AD dementia	Acute single-session pre/post study; 14 AD patients and 14 matched healthy controls.	Single-session manual acupuncture at bilateral LR3 and LI4 for 3 min; healthy-control comparison.	Bilateral aHPC- and pHPC-seeded FC.	After acupuncture, AD patients showed increased hippocampal-subregion FC with regions including the OFC, PHG, STG, and insula, together with decreased FC with the SFG.	Small acute study focused on hippocampal subregions; appears to derive from the same or substantially overlapping cohort as Bai et al. (65), but provides a distinct seed-based analysis.
Bai et al. (65)^#^	AD dementia	Acute single-session pre/post study; 13 AD patients and 14 healthy controls; an additional 100 healthy participants were used to construct the reference NBM-FC map.	Single-session manual acupuncture at bilateral LR3 and LI4 for 3 min; healthy-control comparison.	NBM seed-based FC.	After acupuncture, NBM connectivity with the OFC, lingual gyrus, and ACC decreased in AD patients, whereas NBM–IPL connectivity increased.	Small acute study; appears to arise from the same or substantially overlapping cohort as Fan et al. (64), but provides a distinct NBM-seeded analysis.
Zhan et al. (66)	Mild-to-moderate AD	Randomized longitudinal pilot study; 44 AD patients randomized to acupuncture plus donepezil or donepezil alone, with 22 healthy controls enrolled; final imaging analysis included 20 participants/group.	Both AD groups received donepezil; the treatment group additionally received multi-point acupuncture including GV24, EX-HN3, GV20, EX-HN1, GB12, HT7, KI6, and GB39; 36 sessions over 12 weeks.	fALFF; right-precuneus seed-based FC.	Between-group fALFF and FC differences were reported in precuneus and temporal regions, but these imaging differences did not survive FDR/FWE correction. Within the acupuncture-plus-donepezil group, temporal-region fALFF changes were associated with ADAS-Cog changes after correction.	The design allows evaluation of the additional effect of acupuncture over donepezil, but no sham-acupuncture control was used and the principal between-group imaging findings were based on uncorrected thresholds.
Wei et al. (67)^‖^	Mild-to-moderate AD	Randomized longitudinal study; 60 AD patients randomized to acupuncture plus donepezil or donepezil alone (30/group); rs-fMRI analysis included 11 and 12 patients, respectively.	Both groups received donepezil; the acupuncture group additionally received GV20, EX-HN1, bilateral HT7, PC6, ST36, SP6, and GB39; 30 min/session, 6 days/week for 6 weeks.	Left hippocampal seed-based FC.	Compared with donepezil alone, acupuncture plus donepezil was associated with stronger left hippocampal FC involving temporal, occipital, medial frontal, striatal, and postcentral regions.	Small final imaging sample and no sham-acupuncture control; appears to derive from the same or substantially overlapping 6-week RCT as Wei et al. (68), but provides a distinct hippocampal FC analysis.
Wei et al. (68)^‖^	AD dementia	Randomized longitudinal study; 60 AD patients randomized to donepezil alone or acupuncture plus donepezil (30/group), with 7 healthy controls recruited; final rs-fMRI analysis included 12 drug-group, 11 acupuncture-group, and 6 healthy-control datasets.	Both AD groups received donepezil; the acupuncture group additionally received GV20, EX-HN1, HT7, PC6, ST36, SP6, and GB39; 30 min/session, 6 days/week for 6 weeks.	ICA-defined FPN; Granger-causality EC.	Compared with donepezil alone, acupuncture plus donepezil showed differential directed connectivity involving right insula→left MTG and left precuneus→left MTG; MoCA-B improvement was greater in the acupuncture group.	Randomized longitudinal study examining the additional effect of acupuncture over donepezil; no sham-acupuncture control was used and the healthy-control sample was very small.

This table summarizes clinical acupuncture-related BOLD-fMRI reports included in the primary synthesis. Acute single-session studies reflect short-term brain responses rather than sustained treatment effects. Structural and other non-BOLD neuroimaging findings are mentioned only as related evidence where relevant. Multiple reports arising from the same or potentially overlapping participant cohort were retained when they provided distinct imaging analyses but were not treated as independent participant-level evidence. Acupoint nomenclature follows WHO-standard abbreviations where applicable.

§ Wang et al. (32) and Zhou et al. (33) derive from the same randomized controlled trial; Zhou et al. (33) represents a secondary whole-brain network analysis and was not treated as independent participant-level evidence.

* Feng et al. (39), Bai et al. (40), Chen et al. (38), KI3, and Liu et al. (41) used the same or substantially overlapping small MCI/healthy-control samples and closely related KI3 stimulation paradigms. They were retained as distinct imaging reports but were not treated as independent participant-level evidence.

‡ Wang et al. (59), Liang et al. (60), and Zheng et al. (62) used highly similar participant samples and the same LR3/LI4 stimulation paradigm, suggesting substantial cohort overlap. Wang et al. (63) explicitly reanalyzed the Wang et al. (59) dataset. Ji et al. (61) used a closely related stimulation paradigm but was treated as a separate cohort because the analyzed sample differed demographically.

¶ Chen et al. (43), GV26 and Xu et al. (44) appear to have examined the same six-patient MCI cohort using the same GV26 stimulation paradigm. Chen et al. reported connectivity during needle retention, whereas Xu et al. examined the post-acupuncture resting period; the two reports were therefore retained as distinct temporal analyses but were not treated as independent participant-level evidence.

‖ Wei et al. (67) and Wei et al. (68) appear to derive from the same or substantially overlapping 6-week randomized AD cohort, based on the same sample size, treatment allocation, acupuncture regimen, and treatment duration. They were retained because they report distinct hippocampal functional-connectivity and frontoparietal effective-connectivity analyses, but were not treated as independent participant-level evidence.

# Fan et al. (64) and Bai et al. (65) appear to arise from the same or substantially overlapping acute LR3/LI4 cohort, with closely matched participant samples and the same stimulation paradigm. They were retained because they provide distinct hippocampal-subregion and NBM seed-based connectivity analyses, but were not treated as independent participant-level evidence.

**Table 2:** Critical synthesis of acupuncture-related BOLD-fMRI findings across the clinically defined AD continuum.

Stage	Main regions/networks	Acupuncture-related BOLD-fMRI changes	Cognitive outcomes and associations	Interpretation and limitations
SCD	Prefrontal cortex, hippocampus, PCC/precuneus, insula, temporal cortex, SMN, CON, and parietal network nodes.	A small no-treatment-controlled study reported decreased prefrontal ReHo after acupuncture. Compared with sham acupuncture, longitudinal acupuncture was associated with increased hippocampal FC, altered SMN–CON connectivity, and changes in parietal network topology.	The RCT reported improvement in a composite cognitive score, while the secondary analysis reported improvement in AVLT-H performance and associations between network changes and memory performance.	The available SCD evidence includes regional spontaneous-activity, hippocampal-connectivity, and network-level findings, but remains limited by small samples, different control conditions, and lack of AD biomarker confirmation.
MCI (including aMCI)	DLPFC, ACC, hippocampus and hippocampal subregions, thalamus, insula, temporal cortex, limbic system, parietal regions, and LC-related pathways.	Acute KI3 studies showed short-term FC/EC responses; longitudinal studies reported changes in hippocampal–temporal connectivity, DLPFC-centered ReHo and connectivity, DLPFC–ACC FC/EC, limbic-system connectivity, LC-related pathways, hippocampal-subregion connectivity, and regional ALFF/ReHo.	Several studies reported associations between imaging measures and cognition. DLPFC ReHo or effective-connectivity changes were associated with changes in MoCA or ADAS-Cog, while baseline hippocampal or LC-related connectivity was associated with later memory change. Regional ALFF/ReHo changes were also associated with MoCA change in one study.	Reported findings in MCI span memory- and limbic-related regions, DLPFC–ACC connectivity, locus coeruleus-related pathways, and other large-scale networks. However, study designs, control conditions, acupuncture protocols, treatment duration, study populations, and fMRI methods vary substantially, limiting direct comparison across studies and interpretation of acupuncture specificity.
AD dementia	Hippocampus, parahippocampus, NBM, PCC/precuneus, RSC, temporal cortex, insula, thalamus, orbitofrontal cortex, DMN, FPN, SMN, and visual network.	Most acute LR3/LI4 stimulation studies reported short-term changes in hippocampal–DMN and posterior medial cortical connectivity, with additional bidirectional ALFF changes, enhanced hippocampus–precentral gyrus FC, FPN/VN-specific FC increases or decreases, and bidirectional RSC–FC regulation. More recent acute analyses also reported hippocampal-subregion and NBM seed-based connectivity changes. Two longitudinal cohorts additionally reported fALFF and FC changes involving the precuneus and temporal regions, hippocampal connectivity, and changes in directed connectivity within the FPN.	Most acute studies did not include longitudinal cognitive follow-up. In one 12-week study, temporal-region fALFF changes were associated with changes in ADAS-Cog scores. In the 6-week cohort, greater improvement in MoCA-B was reported with acupuncture plus donepezil than with donepezil alone, although a direct association between imaging and cognitive changes was not reported.	In AD dementia, reported findings mainly involve memory-related pathways and posterior medial cortical regions. Longitudinal cohorts have extended the evidence beyond immediate stimulation responses, but both compared acupuncture plus donepezil with donepezil alone and neither included a sham-acupuncture control. The evidence also remains limited by small samples; one report reanalyzed a previously published dataset, while other studies may involve sample overlap or closely related designs. In one longitudinal study, the main between-group imaging findings did not survive FDR/FWE correction.

This table summarizes acupuncture-related BOLD-fMRI findings across the clinically defined AD continuum rather than providing a formal certainty-of-evidence assessment. Interpretation was based on study design, sample size, control condition, longitudinal outcome assessment, biomarker confirmation, and potential sample overlap. Findings from Wang et al. (32) related to hippocampal volume or structural connectivity were not treated as core BOLD-fMRI evidence in this synthesis. Acute single-session studies should be interpreted as short-term neural responses to acupuncture stimulation rather than evidence of sustained treatment effects.

## 3 Functional network context for acupuncture-related brain responses across the Alzheimer’s disease continuum

Functional abnormalities across the AD continuum extend beyond individual brain regions to altered connectivity within and between large-scale networks (17, 18). Memory impairment is closely linked to the hippocampus and medial temporal lobe, together with posterior cingulate, precuneus, and retrosplenial regions that link medial temporal structures with the default mode network and the posterior medial memory system (19–22). Attention and executive dysfunction also involve prefrontal, cingulo-insular, and frontoparietal systems (23, 24), while locus coeruleus connectivity may also be relevant to attentional function in early AD (25). Acupuncture fMRI studies have reported responses in many of these same regions, including the frontal cortex, insula, thalamus, and parietal cortex (26, 27).

## 4 Clinical functional MRI evidence across the Alzheimer’s disease continuum

### 4.1 Subjective cognitive decline

Subjective cognitive decline (SCD) is characterized by a self-perceived decline in memory or other cognitive abilities despite no clear objective impairment on standard neuropsychological testing (28, 29). Functional imaging studies have reported abnormalities in the default mode network and other memory-related systems, particularly in parietal and temporal regions (28, 30). To date, acupuncture-related fMRI evidence in SCD has included a small 12-week acupuncture-versus-no-treatment study, a 12-week sham-controlled randomized trial, and a secondary analysis of the latter trial. The small study reported decreased prefrontal ReHo after acupuncture compared with no treatment (31). The trial showed increased hippocampal functional connectivity after acupuncture compared with sham acupuncture and also reported changes in hippocampal volume as a structural imaging finding (32). The secondary analysis found significant group-by-time effects on functional connectivity within the sensorimotor network and between the sensorimotor and cingulo-opercular networks, together with changes in parietal nodal properties (33). Together, these studies suggest that acupuncture-related functional changes in SCD may involve regional spontaneous activity, hippocampal connectivity, and broader network organization.

### 4.2 Mild cognitive impairment

Functional imaging studies in MCI have reported abnormalities in spontaneous brain activity, functional connectivity, and network topology, particularly involving the posterior cingulate cortex, parahippocampal gyrus, angular gyrus, and middle temporal gyrus (34–36). Acupuncture-related studies have likewise reported changes in a range of cognition-related regions, including the insula, cingulate cortex, thalamus, and frontal and temporal areas (37). Early fMRI work mainly examined the immediate responses to KI3 stimulation in small, single-session studies. Compared with superficial needling, deep needling was associated with stronger functional connectivity involving the hippocampus, thalamus, and fusiform gyrus, as well as more extensive effective connectivity centered on the DLPFC and hippocampus (38, 39). Other analyses from this early series reported changes in small-world network properties and nodal centrality after deep needling (40), while ReHo and ALFF studies showed regional changes after acupuncture or differences between KI3 and sham stimulation (41, 42). The ReHo findings were less robust, however, as neither the acupuncture main effect nor the group × acupuncture interaction remained significant after multiple-comparison correction (41). A separate six-patient MCI cohort examined GV26 stimulation, with two reports describing altered frontal and parietal functional connectivity during needle retention and after needle withdrawal, respectively (43, 44). Taken together, these early studies mainly reflect short-term responses to stimulation, while differences in needling depth and imaging approach may also have contributed to the variation in reported findings.

Later work in aMCI shifted toward examining brain functional changes over a course of treatment. Across interventions lasting from 4 weeks to 6 months, acupuncture-related changes have been reported in hippocampal–temporal connectivity, DLPFC-centered regional activity and connectivity, and functional interactions involving the insula, thalamus, ACC, and parietal regions (26, 45–48). A randomized sham-controlled study of Siguan acupuncture additionally reported regional ALFF, fALFF, and ReHo changes after treatment (49). The range of findings broadened further in later studies, involving limbic-system connectivity, locus coeruleus-related pathways, hippocampal-subregion connectivity, and regional ALFF and ReHo changes (50–53). Several studies also examined how these imaging findings related to cognitive outcomes. DLPFC ReHo or effective-connectivity changes were associated with changes in MoCA or ADAS-Cog (45, 47), whereas in the locus coeruleus study, later improvement in immediate recall was related to pretreatment connectivity rather than to the magnitude of connectivity change (53). Despite this broader range of findings, substantial differences in acupuncture protocols, control conditions, treatment duration, imaging methods, and study populations still make direct comparison across studies difficult.

### 4.3 Alzheimer’s disease dementia

In AD dementia, functional abnormalities commonly involve the hippocampus, posterior cingulate cortex/precuneus, retrosplenial cortex, and default mode network, all of which are closely related to memory dysfunction (54–58). Earlier acupuncture fMRI studies in this group mainly used a single session of LR3/LI4 stimulation and focused on the immediate brain response. Changes in default mode network activity and connectivity were reported in the posterior cingulate cortex, precuneus, inferior parietal lobule, and middle temporal gyrus, while hippocampal seed-based analysis showed increased connectivity between the left hippocampus and right middle frontal gyrus (59, 60). Other studies found both increases and decreases in ALFF, together with increased hippocampus–precentral gyrus connectivity, and also reported changes in the frontoparietal and visual networks (61, 62). A reanalysis of the Wang et al. (59) dataset using the retrosplenial cortex as the seed region found a different pattern of connectivity change after LR3/LI4 stimulation. In patients with AD, connectivity between the retrosplenial cortex and several posterior medial or limbic-related regions decreased, whereas the corresponding connections increased in healthy controls. By contrast, connectivity with the hippocampus and parahippocampal gyrus increased in the AD group (63). More recent acute analyses from a separate LR3/LI4 cohort reported changes in seed-based connectivity involving hippocampal subregions and the nucleus basalis of Meynert (64, 65). Two longitudinal AD cohorts have also been reported, one of which appears to have contributed two distinct imaging analyses. A 12-week study of acupuncture combined with donepezil reported fALFF and functional connectivity differences involving the precuneus and temporal regions, although the main between-group imaging findings did not survive FDR/FWE correction; temporal fALFF change within the combined-treatment group was associated with change in ADAS-Cog scores (66). In the 6-week cohort, one report described stronger functional connectivity between the left hippocampus and multiple brain regions after acupuncture plus donepezil than after donepezil alone (67), whereas another reported changes in directed connectivity within the frontoparietal network, involving the right insula, left precuneus, and left middle temporal gyrus, together with greater improvement in MoCA-B than with donepezil alone (68). The available studies therefore point mainly to changes involving the hippocampus and posterior medial memory-related regions, while also showing changes in other networks. The earlier evidence largely reflects short-term responses to stimulation, whereas the newer longitudinal cohorts extend these findings to treatment-course changes. However, the lack of sham-acupuncture controls in the longitudinal cohorts, together with small samples and overlapping or reused cohorts, limits the conclusions that can be drawn from the available evidence.

## 5 Discussion

### 5.1 Patterns of brain responses across the AD continuum

The available studies show some differences in the distribution of acupuncture-related BOLD-fMRI findings across clinically defined stages of the AD continuum (69). In SCD, evidence remains limited, with the available studies showing changes in regional spontaneous activity, hippocampal connectivity, and broader network organization; similar alterations in hippocampal and large-scale network organization have also been reported in non-acupuncture imaging studies of SCD (70–72). In MCI, including aMCI, reported changes involve hippocampal and temporal regions, DLPFC–ACC connectivity, locus coeruleus-related pathways, and limbic networks (38, 39, 45, 50, 53, 73). In AD dementia, the earlier acute studies more often involved the hippocampus and posterior medial memory-related regions (59–63), whereas the two longitudinal cohorts also reported changes involving temporal regions, hippocampal connectivity, and frontoparietal effective connectivity (66–68). These differences are difficult to attribute to clinical stage alone. The MCI evidence includes both single-session KI3 studies and longitudinal treatment studies using several fMRI approaches, whereas the earlier AD studies largely used acute LR3/LI4 stimulation and included reports with reused or potentially overlapping samples. The two longitudinal AD cohorts differed again, as both examined acupuncture added to donepezil and neither included a sham-acupuncture control. Across stages, the studies also varied in acupuncture protocols, control conditions, sample size, imaging methods, and follow-up. Functional connectivity itself changes across MCI and AD in the broader imaging literature (74–77), further limiting the extent to which the differences observed here can be interpreted as stage-related.

### 5.2 Broad network responses and memory-related circuits

The hippocampus and posterior medial memory-related regions recur throughout the studies reviewed. Acupuncture-related changes have been reported in the hippocampus, posterior cingulate cortex, precuneus, retrosplenial cortex, prefrontal regions, and other limbic structures. Many of these regions overlap with the default mode network and the medial temporal–posterior medial memory system, both of which are vulnerable in AD (78, 79). ALFF and ReHo reflect regional aspects of spontaneous BOLD activity, whereas functional connectivity and network analyses characterize interactions among distributed brain regions, capturing different levels of functional organization (80–83).

The reported changes are not uniformly directional, with both increased and decreased brain activity or functional connectivity observed across studies. The broader imaging literature similarly shows both increased and decreased connectivity across MCI and AD, and longitudinal changes in hippocampal network connectivity have also been linked to cognitive decline (84–86). This variation makes it difficult to interpret the direction of an imaging change without considering the measure used and the underlying functional state of the network.

Acupuncture provides somatosensory input that can engage distributed cortical and subcortical regions (87, 88). Because cognition-related networks such as the DMN, SN, and FPN are involved in cognitive control and salience processing (89, 90) and show altered connectivity in MCI and AD (91, 92), the BOLD-fMRI response observed after acupuncture may also depend on the functional state of these networks before stimulation. The association between pretreatment locus coeruleus connectivity and later memory improvement in aMCI further suggests that baseline network state may be relevant to subsequent cognitive response (53). Increases or decreases in brain activity or functional connectivity do not by themselves indicate functional improvement or decline, and some changes may reflect compensatory processes (93). Whether these imaging changes are accompanied by cognitive improvement or relate to AD pathology remains uncertain.

### 5.3 Acupuncture intervention and control design

The early MCI studies mainly examined a single session of KI3 stimulation, comparing deep with superficial needling, whereas earlier studies in AD dementia focused on the immediate response to LR3/LI4 stimulation. The SCD trial and several studies in aMCI, by contrast, used multi-point prescriptions over several weeks. Two longitudinal AD cohorts also used multi-point acupuncture over 6 or 12 weeks in addition to donepezil (66–68). The studies differed not only in acupoint selection, but also in needling depth, stimulation method, retention time, treatment frequency, and treatment duration. Stimulation intensity and de qi can influence sensory and central responses (94), and variation in these features may also contribute to differences in the imaging findings. The Standards for Reporting Interventions in Clinical Trials of Acupuncture (STRICTA) recommend detailed reporting of the acupuncture rationale, needling procedures, treatment regimen, and practitioner background (95, 96).

The included studies also used different comparison groups, including sham acupuncture, superficial needling, non-acupoint needling, wait-list controls, healthy participants, and medication-only controls. Sham, superficial, and non-acupoint needling can still provide somatosensory input and may themselves elicit physiological responses (97, 98). Wait-list controls can show whether changes occur over time but do not account for treatment contact or expectation, whereas healthy-control comparisons can reveal differences in brain responses between patients and healthy individuals without establishing acupuncture specificity (99). The two longitudinal AD cohorts compared acupuncture plus donepezil with donepezil alone, but neither included a sham-acupuncture control (66–68). Sham-controlled comparisons may also be influenced by the physiological effects of the sham procedure itself, as well as by blinding and participant expectations (100, 101). With these control designs, the current fMRI evidence is better suited to describing acupuncture-related brain changes than to establishing effects specific to needling or acupoint selection.

### 5.4 Relationship between BOLD-fMRI findings and cognitive outcomes

Changes in functional connectivity were associated with cognitive change in both the SCD secondary analysis and the aMCI trial, although the networks involved differed (33, 45). In the locus coeruleus study, later improvement in immediate recall was related to pretreatment connectivity (53), suggesting that baseline network state may also be relevant to cognitive response. Most earlier acupuncture fMRI studies in AD dementia assessed only the immediate response to a single acupuncture session and did not follow cognition over time, so it remains unclear whether these acute BOLD changes are related to later cognitive change. The two longitudinal AD cohorts also reported cognitive outcomes alongside imaging changes. In the 12-week study, temporal-region fALFF changes within the acupuncture-plus-donepezil group were associated with changes in ADAS-Cog scores (66). In the 6-week cohort, frontoparietal effective-connectivity changes were reported together with greater improvement in MoCA-B in the acupuncture-plus-donepezil group than in the donepezil-only group (68). With only two longitudinal cohorts, however, it remains unclear whether the imaging changes are consistently related to cognitive improvement. Longitudinal neuroimaging studies outside the acupuncture literature also show that both increases and decreases in functional connectivity can accompany cognitive decline (84). Brain activity may also be associated with preserved cognitive performance despite underlying AD pathology (102), while resting-state functional connectivity does not necessarily parallel clinical change (103).

### 5.5 Biomarker status and future directions

Most acupuncture BOLD-fMRI studies grouped participants by clinical diagnosis, with little confirmation of amyloid-β or tau pathology. The same clinical diagnosis may therefore include individuals with different degrees of underlying AD pathology, particularly in SCD and MCI, where clinical symptoms do not necessarily correspond to the same biological stage (104, 105). Biomarkers are increasingly incorporated into AD clinical trials to characterize disease pathology and treatment-related changes (106). Including this information in acupuncture studies would help clarify whether differences in BOLD-fMRI findings are related to clinical stage, pathological burden, or both. Baseline network organization may also influence the relationship between AD pathology and cognitive decline (107). Interpretation of BOLD-fMRI is further complicated by the vascular changes that accompany AD. The BOLD signal depends on cerebral blood flow and neurovascular coupling, both of which can be altered as the disease progresses (108, 109). Acupuncture-related BOLD changes may therefore reflect both neural and vascular factors. Perfusion imaging and structural MRI, considered alongside AD biomarkers, may help distinguish these influences and relate the functional changes more closely to the underlying disease process (110, 111).

Acquisition and preprocessing procedures also varied across studies, most samples were small, and follow-up was often limited. Methodological limitations were considered narratively in this review rather than through a formal risk-of-bias assessment, which also limits the extent to which differences in study quality can be incorporated into the interpretation of the evidence. These factors make it difficult to determine whether differences in the reported findings reflect clinical stage, acupuncture protocol, or analytic choices. Prespecified imaging hypotheses and more complete reporting of acquisition, motion control, preprocessing, and longitudinal assessment would improve comparability across studies (112–116). Longer follow-up is also important for determining whether the observed imaging changes persist and whether they are accompanied by changes in cognition or daily functioning (117, 118). The main methodological considerations are summarized in Table 3.

**Table 3:** Methodological considerations for future acupuncture BOLD-fMRI studies.

Domain	Priority	Why it matters	Suggested reporting
Disease definition	Report clinical diagnostic criteria; when available, report amyloid-β, tau, or AT(N) biomarker status.	Clinically defined SCD, MCI (including aMCI), and AD dementia may include individuals with different underlying AD pathological profiles.	Specify the clinical diagnostic category and whether underlying AD pathology was biomarker-confirmed.
Acupuncture protocol	Describe acupoints, laterality, stimulation method, insertion depth, retention time, treatment frequency, treatment course, de qi, and practitioner qualification.	Different acupoints, stimulation intensities, and de qi states may be associated with different BOLD-fMRI responses.	Follow the STRICTA framework and avoid vague descriptions such as “multi-point acupuncture” without operational details.
Control condition	Clearly define sham acupuncture, superficial needling, non-acupoint needling, wait-list control, healthy-control comparison, or medication-only control.	Sham or superficial needling may still produce somatosensory input and should not be assumed to be physiologically inert. Medication-only controls can assess the additional effect of acupuncture over background treatment but do not control for nonspecific acupuncture-related effects.	Unless specificity is supported by adequate sham-controlled evidence, describe findings as acupuncture-related brain functional changes.
fMRI acquisition and preprocessing	Report scanning parameters, head-motion thresholds, nuisance regression, temporal filtering, smoothing, multiple-comparison correction, and sensitivity analyses.	In AD populations, BOLD signals may be influenced by neurovascular factors, head motion, and analytic choices.	Define primary imaging hypotheses when possible and distinguish confirmatory analyses from exploratory analyses.
Outcome association	Report cognitive outcomes alongside imaging findings and examine brain–cognition associations longitudinally where possible.	Changes in BOLD-fMRI measures do not by themselves indicate cognitive improvement; imaging and cognitive changes need to be evaluated together.	Distinguish immediate BOLD responses from longer-term imaging changes, and report whether imaging and cognitive outcomes change together over time.
Evidence synthesis	Identify sample overlap, distinguish acute from longitudinal designs, and clarify whether findings come from BOLD-fMRI or other multimodal evidence.	One AD dementia LR3/LI4 report reanalyzed a previously published dataset, while other studies may include overlapping participants or use closely related designs, limiting the independence of the evidence.	Avoid counting overlapping cohorts as mutually independent evidence.

This table summarizes key methodological issues for future acupuncture BOLD-fMRI studies across the clinically defined AD continuum, including disease definition, acupuncture reporting, control design, fMRI analysis, brain-behavior interpretation, and evidence independence.

## 6 Conclusion

Across the available acupuncture BOLD-fMRI literature, studies in SCD have mainly reported changes in regional spontaneous activity, hippocampal connectivity, and network organization, whereas findings in MCI, including aMCI, have involved memory- and limbic-related regions, DLPFC–ACC connectivity, and locus coeruleus-related pathways. In AD dementia, reported changes have more often involved the hippocampus and posterior medial memory-related regions, with earlier studies mainly examining short-term responses to acupuncture and two longitudinal cohorts extending these observations over a treatment course. These distributions should not be interpreted as stage-specific effects, because the studies differ substantially in acupuncture protocols, control conditions, imaging methods, sample size, biomarker status, and follow-up. More comparable longitudinal studies, particularly those incorporating appropriate control conditions, AD biomarkers, and cognitive follow-up, are needed to determine whether the observed differences in imaging findings are related to clinical stage and whether these changes persist alongside cognitive change.

## Glossary

ACC: Anterior cingulate cortex
AD: Alzheimer’s disease
ADAS-Cog: Alzheimer’s disease assessment scale–cognitive subscale
ALFF: Amplitude of low-frequency fluctuations
aMCI: Amnestic mild cognitive impairment
CON: Cingulo-opercular network
DLPFC: Dorsolateral prefrontal cortex
DMN: Default mode network
EC: Effective connectivity
FC: Functional connectivity
FPN: Frontoparietal network
FWE: Family-wise error
ICA: Independent component analysis
ITG: Inferior temporal gyrus
LC: Locus coeruleus
MCI: Mild cognitive impairment
MFG: Middle frontal gyrus
MoCA: Montreal cognitive assessment
MTG: Middle temporal gyrus
OFC: Orbitofrontal cortex
PCC: Posterior cingulate cortex
ReHo: Regional homogeneity
RSC: Retrosplenial cortex
RSFC: Resting-state functional connectivity
SCD: Subjective cognitive decline
SMN: Sensorimotor network
FDR: False discovery rate
ROI: Region of interest
SCG: Subcallosal cingulate gyrus
VN: Visual network
aHPC: Anterior hippocampus
aPFC: Anterior prefrontal cortex
fALFF: Fractional amplitude of low-frequency fluctuations
IPL: Inferior parietal lobule
NBM: Nucleus basalis of Meynert
PHG: Parahippocampal gyrus
pHPC: Posterior hippocampus
SFG: Superior frontal gyrus
STG: Superior temporal gyrus
VMPFC: Ventromedial prefrontal cortex
