# Supplementary material for: Acupuncture-related brain network findings across clinical stages of the Alzheimer’s disease continuum: a critical review of functional MRI evidence
Source: Front Neurol. 2026 Oct 28;17:1977763. doi: 10.3389/fneur.2026.1977763 (PMC13752398; doi:10.3389/fneur.2026.1977763)
Supplement: Supplementary file 1 [file Table_1.DOCX]

**Supplementary Table S1. Complete database-specific search strategies used in the literature search**

| **Database** | **Date last searched** | **Complete search strategy** |
| --- | --- | --- |
| ****PubMed**** | 14 October 2026 | ("Alzheimer Disease"[Mesh] OR Alzheimer*[Title/Abstract] OR "subjective cognitive decline"[Title/Abstract] OR "subjective cognitive impairment"[Title/Abstract] OR "mild cognitive impairment"[Title/Abstract] OR "amnestic mild cognitive impairment"[Title/Abstract] OR "amnestic cognitive impairment"[Title/Abstract] OR aMCI[Title/Abstract]) AND ("Acupuncture Therapy"[Mesh] OR "Electroacupuncture"[Mesh] OR acupuncture[Title/Abstract] OR electroacupuncture[Title/Abstract] OR "electro-acupuncture"[Title/Abstract] OR "manual acupuncture"[Title/Abstract]) AND ("functional magnetic resonance imaging"[Title/Abstract] OR fMRI[Title/Abstract] OR "BOLD-fMRI"[Title/Abstract] OR BOLD[Title/Abstract] OR "blood oxygen level-dependent"[Title/Abstract] OR "resting-state fMRI"[Title/Abstract] OR "resting state fMRI"[Title/Abstract] OR rs-fMRI[Title/Abstract] OR "functional connectivity"[Title/Abstract] OR "effective connectivity"[Title/Abstract] OR "regional homogeneity"[Title/Abstract] OR ReHo[Title/Abstract] OR "amplitude of low-frequency fluctuation"[Title/Abstract] OR "amplitude of low frequency fluctuation"[Title/Abstract] OR ALFF[Title/Abstract] OR fALFF[Title/Abstract] OR "independent component analysis"[Title/Abstract] OR ICA[Title/Abstract] OR "graph theory"[Title/Abstract] OR "brain network"[Title/Abstract] OR "brain networks"[Title/Abstract] OR "default mode network"[Title/Abstract] OR hippocamp*[Title/Abstract]) |
| ****Web of Science Core Collection**** | 14 October 2026 | **#2:** TS=(Alzheimer* OR "subjective cognitive decline" OR "subjective cognitive impairment" OR "mild cognitive impairment" OR "amnestic mild cognitive impairment" OR "amnestic cognitive impairment" OR aMCI) **#4:** TS=(acupuncture OR electroacupuncture OR "electro-acupuncture" OR "manual acupuncture") **#5:** TS=("functional magnetic resonance imaging" OR fMRI OR "BOLD-fMRI" OR BOLD OR "blood oxygen level-dependent" OR "resting-state fMRI" OR "resting state fMRI" OR rs-fMRI OR "functional connectivity" OR "effective connectivity" OR "regional homogeneity" OR ReHo OR "amplitude of low-frequency fluctuation" OR "amplitude of low frequency fluctuation" OR ALFF OR fALFF OR "independent component analysis" OR ICA OR "graph theory" OR "brain network" OR "brain networks" OR "default mode network" OR hippocamp*) **Final search:** #2 AND #4 AND #5 |
| ****China National Knowledge Infrastructure (CNKI), Search 1**** | 14 October 2026 | **主题：** (阿尔茨海默病 + 阿尔茨海默症 + 老年性痴呆 + 轻度认知障碍 + 遗忘型轻度认知障碍 + 遗忘性轻度认知障碍 + 主观认知下降 + 主观认知衰退) **AND 主题：** (针刺 + 针灸 + 电针) **AND 主题：** (功能磁共振 + 功能磁共振成像 + 静息态功能磁共振 + 静息态功能磁共振成像 + fMRI) |
| ****China National Knowledge Infrastructure (CNKI), Search 2**** | 14 October 2026 | **主题：** (阿尔茨海默病 + 阿尔茨海默症 + 老年性痴呆 + 轻度认知障碍 + 遗忘型轻度认知障碍 + 遗忘性轻度认知障碍 + 主观认知下降 + 主观认知衰退) **AND 主题：** (针刺 + 针灸 + 电针) **AND 主题：** (ALFF + fALFF + ReHo + 低频振幅 + 低频振荡振幅 + 局部一致性 + 功能连接 + 有效连接 + 脑功能网络 + 功能网络 + 默认模式网络) |

**Note:** In CNKI, searches were conducted in the Topic field. The “+” operator in the CNKI search history represents OR between terms within the same field.
